# A New Caustic

**Published:** 1867-02

**Authors:** P. W. Ellsworth

**Affiliations:** Hartford, Connecticut


*A New Caustic.* By P. W. Ellsworth, M. D., of Hartford, Connecticut.

Will you permit me to call the attention of the profession to a new medical agent, or at least a new application of an agent in pretty general use, but whose properties are not yet fully understood, viz., sun-light.

It has been a great desideratum in the profession to devise some method of removing nævi, marks, discolorations, moles and other diseased conditions of the skin, whether natural or acquired, without subjecting the patient to the knife, or leaving a cicatrix quite as repulsive as the original disease. A Mr. Augustus Barnes, a true Yankee, but not a member of our profession, thinks he has hit upon such an agent—first experimenting upon himself upon a mole; and I am much inclined to believe he has made a valuable discovery.

He uses a lens of two and three inch diameter, condensing the rays upon the object to be removed, and going over the whole, if not more than three in surface, at one sitting. Mr. Barnes, who is a very pleasant, agreeable gentleman, called on me a few weeks ago, and introduced the subject. At first it did not strike my fancy, as I supposed the pain would be equally severe with other caustics, and the effects no way superior. However, I witnessed his operation with fairness, and with interest, and am disposed to give him considerable credit, and believe his discovery in scientific hands will be made more generally useful than even the inventor believes. I have seen one gentleman, who had a nævus on his face, extending from the eye to below the mouth, and involving the lower eyelid to the very edge, and covering four or five square inches of surface; it was of a deep cherry-red color, approaching purple, and covered with knobs of condensed tissue, an eighth of an inch high. This nævus could be seen as far off as the color of the face. After two applications the spot has nearly disappeared, the skin generally having the hue of a surface blistered some days previously, and it is now nearly well. Some portions were absolutely like normal skin, and entirely colorless. Every knob was gone, and where stood one of the largest, and where the rays were longest condensed, was a perfectly healthy-looking cutis. I do not consider this man as absolutely well, but so much better than he would have been under any known agent, that I must confess my hopes have been considerably raised. As a deformity, or rather as a mark, this man can be considered practically cured, although there is at present the appearance stated, but which does not especially draw attention. I would add, that the rays were condensed with excellent success, even on the very edge of the lid. Mr. Barnes applied his caustic not only to discolorations, but to small tumors involving the surface of the skin, to lupus and ulcerations. He claims to have produced a true and healthy skin of the surface affected by ichthyosis.

How the light, as a caustic, operates differently from other agents, it may be difficult to say, but it has struck me that as the rays are possessed of powerful bleaching properties, it is possible this principle may be brought into play. If the pigment is destroyed, and the secreting power of the corpus mucosum changed, there may be an alteration in the color, without impairment of the cutis vera, which latter seems in all cases to have remained uninjured.

Nor is the pain as severe as we might apprehend, as it is confined at each instant to a very minute point, and therefore must be less perceptible than when diffused over a large surface. Patients at any rate submit very readily and without the use of anæsthetics. I would here suggest, that probably we may not find in this remedy for the lead-colored skin produced by light acting on nitrate of silver. It would be less likely to cure than when the discoloration was from some other cause, since it is the effect of light. There is this difference, moreover, that in the nitrate of silver stain the whole skin may be impregnated, while in nævi the discoloration is confined to some particular tissue or layer. I strongly suspect the skin of the negro might be changed to some degree more probably than in case of coloring with nit. silver.

As to the removal of lupus and small cancers, we may well entertain grave doubts. But as there is no proof that cancer in its incipiency is not a local disease, it would be wrong to pronounce too hasty judgment. I intend making further experiments with this agent, and hope others of the profession will do the same, and give the results to the public. I regret that Mr. Barnes talks of getting a patent, but he is a non-medical man, he thinks his idea, if valuable, should be paid for in some way. He can hardly be compensated in that matter; but if the discovery is as useful as there is reason to hope, he deserves the thanks of the profession.—*Phil. Med. and Surg. Reporter.*

